# Clinical Significance of *POU5F1P1* rs10505477 Polymorphism in Chinese Gastric Cancer Patients Receving Cisplatin-Based Chemotherapy after Surgical Resection

**DOI:** 10.3390/ijms150712764

**Published:** 2014-07-18

**Authors:** Lili Shen, Mulong Du, Chun Wang, Dongying Gu, Meilin Wang, Qi Zhang, Tingting Zhao, Xunlei Zhang, Yongfei Tan, Xinying Huo, Weida Gong, Zhi Xu, Jinfei Chen, Zhengdong Zhang

**Affiliations:** 1 Department of Oncology, Nanjing First Hospital, Nanjing Medical University, 68 Changle Road, Nanjing 210006, China; E-Mails: shenlilimao2012@163.com (L.S.); wangchun850526@hotmail.com (C.W.); gu_dongying@sina.com (D.G.); jhcxy1225@126.com (Q.Z.); zhaoting19900912@163.com (T.Z.); huoxinying2008@126.com (X.H.); michelle.xuzhi@gmail.com (Z.X.); 2 Department of Environmental Genomics, Jiangsu Key Laboratory of Cancer Biomarkers, Prevention and Treatment, Cancer Center, Nanjing Medical University, 818 East Tianyuan Road, Nanjing 211166, China; E-Mails: dumulong@163.com (M.D.); meilin_w@163.com (M.W.); 3 Department of Genetic Toxicology, the Key Laboratory of Modern Toxicology of Ministry of Education, School of Public Health, Nanjing Medical University, 818 East Tianyuan Road, Jiangning District, Nanjing 211166, China; 4 Department of Oncology, Nantong Tumor Hospital, Nantong 226000, 30 Tongyang North Road, China; E-Mail: yjsnj1231@hotmail.com; 5 Department of Surgery, Yixing People’s Hospital, 75 Tongzhenguan Road, Yixing 214200, China; E-Mail: shelly37319@126.com; 6 Department of General Surgery, Yixing Tumor Hospital, 45 Dongshan East Road, Yixing 214200, China; E-Mail: gongweida2010@gmail.com

**Keywords:** gastric cancer, *POU5F1P1* rs10505477 polymorphism, single nucleotide polymorphism (SNP), cisplatin-based chemotherapy

## Abstract

This study aimed to investigate the association between *POU class5 homeobox 1 pseudogene 1* gene (*POU5F1P1*) rs10505477 polymorphism and the prognosis of Chinese gastric cancer patients, who received cisplatin-based chemotherapy after surgical resection. *POU5F1P1* rs10505477 was genotyped using the SNaPshot method in 944 gastric cancer patients who received gastrectomy. The association of rs10505477 G > A polymorphism with the progression and prognosis in gastric cancer patients was statistically analyzed using the SPSS version 18.0 for Windows. The results reveal that rs10505477 polymorphism has a negatively effect on the overall survival of gastric cancer patients in cisplatin-based chemotherapy subgroup (HR = 1.764, 95% CI = 1.069–2.911, *p* = 0.023). Our preliminary study indicates for the first time that *POU5F1P1* rs10505477 is correlated with survival of gastric cancer patients who receving cisplatin-based chemotherapy after gastrectomy. Further studies are warranted to investigate the mechanism and to verify our results in different populations.

## 1. Introduction

As the fourth most common cancer and the second leading cause of cancer-related death worldwide, gastric cancer (GC) contributes to a significant burden of disease, particularly in economically less-developed countries [1,2]. Although both its morbidity and mortality have been declining in the latest decade [2], GC patients still have a poor 5-year survival rate [3]. In recent years, several studies have demonstrated that GC is a stem cell disease [4,5,6,7]. This viewpoint offers us a brilliant insight to understand the molecular mechanism of gastric cancer and to identify new diagnostic and therapeutic targets for gastric cancer. As far as is known, tumor stem cells are under control of numerous regulatory factors, among them transcription factors should be considered as one of the most important regulatory factors [8]. Hence, it is warranted to identify potential markers of gastric cancer stem cell and related regulatory factors. Furthermore, exploration of the genetic variants in regulatory factor genes involved in the progress and prognosis of gastric cancer is also very important.

*POU5F1* (also called OCT4 or OCT3) is a central gene in the regulation of stem cell pluripotency [9,10,11]. Some investigators [12,13] found that the over-expression of *POU5F1* is significantly associated with the invasion and metastasis of GC. *POU5F1P1* (also called *POU5F1B*) gene is classified as a highly homologous pseudo-gene of *POU5F1* [14]. Panagopoulos and his colleagues have reported that *POU5F1P1* produces a protein with similar function to *POU5F1* [15]. So we hypothesize that the variants of *POU5F1P1* may play a part in the tumorigenesis and progression of gastric cancer through influencing the function of *POU5F1*. *POU5F1P1* is located in 8q24.21 region 3. Preliminary GWAS (Genome Wide Association Studies) and follow-up studies were carried out to reveal functional signal nucleotide polymorphisms (SNPs) of *POU5F1P1* involved in cancer. Pal *et al.* [16] have found strong evidence of the association of *POU5F1P1* rs871135 G > T polymorphism with prostate cancer and Wei *et al.* [17] revealed *POU5F1P1* rs7014346 G > A polymorphism was significantly associated with breast cancer. Most studies of *POU5F1P1* gene polymorphism concern the loci rs10505477; studies have shown that the rs10505477 C > T polymorphism plays an important role in the oncogenesis and progression of colorectal cancer (CRC) [18,19,20,21,22], but not in ovarian cancer [23]. However the association of rs10505477 with GC is poorly understood. In 2011, Paul *et al.*’s research first detected that there was no significant association of rs10505477 with upper gastrointestinal cancer in Caucasians [24]. As genetic variation is geographically structured, an allele tends to become more frequent in one population but not in another. Therefore we performed this genotyping study to see if the relationship between gastric cancer and *POU5F1P1* rs10505477 G > A polymorphism in a Chinese Han population would be consistent with Paul’s study.

## 2. Results

### 2.1. Patients’ Characteristics

Nine-hundred and nine samples were included in this study after excluding those patients with failed genotyping. The patients’ characteristics and clinical information are summarized in Table 1. All patients received surgical resections, among which 291 had undergone chemotherapy. There were 700 males (77.0%) and 209 females (23.0%), with the median age of 61 years ranging from 28 to 83 years. In the follow-up period of 119 months (last follow-up in March 2009), we observed that a sum of 418 (46.0%) patients died. The maximum survival time was 119.0 months and the median survival time was 70.0 months. Our study confirmed that clinicopathologic characteristics, including tumor size, histological types, depth of invasion, lymph node metastasis, and TNM (Tumor, Node, Metastasis) stage were closely related to survival time (log-rank *p* < 0.05). Specifically, patients with tumor size > 5 cm (median survival time (MST), 51 months) had a 40.9% significantly higher risk of death (HR = 1.409, 95% CI = 1.161–1.710) compared with those with tumor size ≤ 5 cm (MST, 74 months), and the diffuse-type gastric cancer patients (MST, 52 months) had a 45.3% significantly higher risk of death (HR = 1.453, 95% CI = 1.189–1.776) than those intestinal-type patients (MST, 77 months). In addition, as the depth of invasion and TNM stage increased, the risk of death for gastric cancer showed a significant increase in a dose-dependent manner (log-rank *p* < 0.001).

### 2.2. Associations of POU5F1P1 rs10505477 with Prognosis of Gastric Cancer (GC) Patients

Among 944 GC patients with complete clinical follow-up information, rs10505477 was successfully genotyped in 909 specimens. The frequency of each genotype was 34.7% (315 specimens) for the GG variant, 47.7% (434 specimens) for the GA variant, 17.6% (160 specimens) for the AA variant. Cox regression analysis was used to detect the association of rs10505477 polymorphism with gastric cancer survival in various genetic models. Regrettably, there was no association between *POU5F1P1* rs10505477 G > A polymorphisms and the survival of GC patients in either genotype models (log-rank *p* = 0.185 for co-dominant model; log-rank *p* = 0.177 for dominant model; log-rank *p* = 0.478 for recessive model; as present in Table 2).

**Table 1:** Association between clinicopathological features and survival of gastric cancer.

Variables		Patients, *n* = 909		Deaths, *n* = 418		MST (Months)		log-Rank *p*		HR (95% CI)	
Age (years)											
≤60		429		195		97		0.354		1.000	
>60		480		223		62				1.095 (0.903–1.327)	
Sex											
Male		700		320		74		0.57		1.000	
Female		209		98		67				1.067 (0.851–1.338)	
Tumor size											
≤5 cm		564		236		74		<0.001		1.000	
>5 cm		345		182		51				1.409 (1.616–1.710)	
Location											
Non-Cardia		601		280		70		0.371		1.000	
Cardia		308		138		77				0.912 (0.744–1.118)	
Histological types											
Intestinal		387		149		77		<0.001		1.000	
Diffuse		518		266		52				1.453 (1.189–1.776)	
Others		4		3		11				2.732 (0.871–8.571)	
Differentiation ^a^											
Well-to-moderate		297		125		80		0.49		1.000	
Poorly		472		228		62				1.158 (0.931–1.441)	
Mucinous/signet-ring cell		65		32		62				1.202 (0.815–1.772)	
Others		75		33		67				0.986 (0.671–1.448)	
Depth of invasion ^b^											
T1		177		57		N/A^1^		<0.001		1.000	
T2		130		56		78				1.452(1.004–2.101)	
T3		6		3		70				1.427(0.447–4.559)	
T4		578		291		52				1.839(1.383–2.446)	
Lymph node metastasis ^c^											
N0		359		128		N/A^1^		<0.001		1.000	
N1/N2/N3		529		277		48				1.731 (1.403–2.136)	
Distant metastasis											
M0		891		407		74		0.296		1.000	
M1		16		9		40				1.417 (0.732–2.743)	
TNM stage											
I		239		80		N/A^1^		<0.001		1.000	
II		195		77		N/A^1^				1.241 (0.907–1.698)	
III		447		244		41				1.993 (1.547–2.568)	
IV		22		11		47				1.823 (0.970–3.424)	
Chemotherapy											
No		618		293		62		0.344		1.000	
Yes		291		125		98				0.904 (0.734–1.115)	
Chemotherapy regimes											
l-OHP		109		38		60		0.082		1.000	
DDP		179		89		51				1.398 (0.954–2.048)	
Smoking											
Non-smoker		833		386		67		0.432		1.000	
Smoker		76		32		97				0.866 (0.604–1.243)	
Drinking											
Non-drinker		850		389		70		0.691		1.000	
Drinker		59		29		63				1.079 (0.740–1.574)	

Abbreviations: MST, median survival time; HR, hazard ratio; CI, confidence interval; TNM, Tumor, Node and Metastasis; l-OHP, oxaliplatin; DDP, cisplatin. ^a^ Partial data were not available, and statistics were based on available data; ^b^ The information about the depth of invasion was not available for two patients; invaded depth of tumor was classified according to the criteria of American Joint Commission on Cancer (AJCC) 7th; ^c^ Lymph nodes were staged according to tumor-node-metastasis classification of the 7th edition of AJCC in which the number of lymph nodes with a metastasis of 1, 2, 3, 6 and 7 were classified as N1, N2 and N3, respectively. N/A^1^, Mean the median survival time could not be measured.

**Table 2:** Association between rs10505477 polymorphism and overall survival of gastric cancer.

Genetic Model	Genotypes	Patients	Deaths	MST (Months)	log-Rank *p*	HR (95% CI) *
Codominant model	GG	315	136	77	0.185	1.000
	GA	434	215	60		1.200 (0.968–1.488)
	AA	160	67	69		1.014 (0.757–1.359)
Dominant model	GG	315	136	77	0.177	1.000
	GA/AA	594	282	63		1.150 (0.937–1.411)
Recessive model	GG/GA	749	351	67	0.478	1.000
	AA	160	67	N/A^1^		0.910 (0.701–1.182)

Abbreviations: MST, median survival time; HR, hazard ratio; CI, confidence interval; * Hazard Ratio (HR) adjusted for age, sex, Tumor, Node and Metastasis (TNM) stage; N/A^1^, Mean the median survival time could not be measured.

We further assessed the association of *POU5F1P1* rs10505477 polymorphisms with gastric cancer survival by stratified analysis of tumor size, tumor site, histological type, depth of invasion, lymph node metastasis, distant metastasis, TNM stage and chemotherapy. The results are shown in Table 3. In the different subgroups of patients, there was no significant association between genotypes and survival of GC patients in any genetic models.

**Figure 1:**
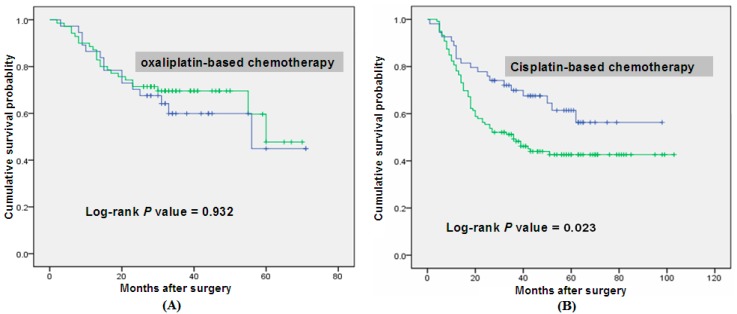
Overall survival curve in relation to *Pit-Oct-Unic Class 5 Homeobox 1 Pseudogene 1* Gene (*POU5F1P1*) rs10505477 polymorphism in gastric cancer patients in dominant model. (**A**) demonstrates that when compared with the GG genotype, GA/AA genotypes had nodifference on overall survival in oxaliplatin-based chemotherapy subgroup (*p* = 0.932); (**B**) demonstrates that the GA/AA genotypes had negative effects on overall survival in the subgroups of patients receiving cisplatin-based chemotherapy (*p* = 0.023).

Then we stratified patients by chemotherapy regimens (based on cisplatin and oxaliplatin) and performed the Cox regression, Kaplan–Meier survival curves and the log-rank test to evaluate the association of rs10505477 genotypes with survival in stratified patients. Exhilaratingly, in dominant models, GA/AA genotypes had negative effect on overall survival of patients receiving chemotherapy based on cisplatin (HR = 1.764, 95% CI = 1.069–2.911, *p* = 0.023, Table 4). But no similar results were found in subgroup with chemotherapy based on oxaliplatin (l-OHP). And the survival curve was shown in Figure 1. It demonstrated that compared with the G allele, the A allele was a risk factor for the prognosis of these patients having chemotherapy based on cisplatin (CDDP).

Finally, stepwise Cox regression analysis was performed to obtain the association between included demographic characteristics, clinical features, the rs10505477 SNP and gastric cancer patients’ survival. As shown in Table 5, one variable (regimens: oxaliplatin *vs.* cisplatin) was included in the Cox regression model with a significance level for *p* < 0.05 entering and *p* > 0.10 for removing a variable (*p* = 0.048).

**Table 3:** Stratified analysis of association between rs10505477 polymorphism and overall survival of gastric cancer.

Variables	Genotypes (Dominant Model)		HR (95% CI) ^a^	*p* Heterogeneity
	GG	GA/AA		
Total (*n* = 909)	315	594	1.150 (0.937–1.411)	0.177
Tumor size				
≤5 cm	202	362	1.248 (0.949–1.640)	0.112
>5 cm	113	232	0.989 (0.726–1.348)	0.945
Tumor site				
Non-Cardia	207	394	1.226 (0.951–1.581)	0.116
Cardia	108	200	1.038 (0.733–1.469)	0.835
Lauren classification				
Intestinal type	145	242	0.996 (0.715–1.387)	0.98
Diffuse type	170	352	1.224 (0.941–1.593)	0.132
Differentiation				
Well to moderate	114	183	0.988 (0.689–1.418)	0.949
Poorly	160	312	1.102 (0.835–1.454)	0.494
Mucinous/signet-ring cell	19	46	2.075 (0.850–5.065)	0.109
Others	22	53	1.674 (0.754–3.718)	0.206
Depth of invasion				
T1	66	111	1.072 (0.634–1.813)	0.796
T2	47	83	1.502 (0.842–2.680)	0.168
T3	2	4	0.627 (0.138–2.837)	0.544
T4	194	384	1.115 (0.872–1.425)	0.385
Lymph node metastasis				
N0	137	222	1.181 (0.842–1.655)	0.335
N1/N2/N3	170	369	1.135 (0.877–1.469)	0.336
Distant metastasis				
M0	309	582	1.130 (0.914–1.398)	0.259
M1	6	10	1.544 (0.721–3.305)	0.264
TNM stage				
I	93	149	1.238 (0.789–1.942)	0.352
II	64	195	1.032 (0.640–1.664)	0.896
III	150	299	1.080 (0.826–1.412)	0.574
IV	8	14	2.301 (0.600–8.818)	0.224
Chemotherapy				
No	208	410	1.144 (0.893–1.466)	0.286
Yes	107	184	1.414 (0.792–1.645)	0.478

Abbreviations: HR, hazard ratio; CI, confidence interval; ^a^ Hazard Ratio (HR) adjusted for age, sex.

**Table 4:** Association between the dominant model of rs10505477 and overall survival of gastric cancer among chemotherapy regimen subgroup.

Chemotherapy Based on l-OHP					
Genotype	Patients, *n* =108	Deaths, *n* = 38	MST (Months)	log-Rank *p*	HR (95% CI) ^a^
GG	39	14	55	0.932	1.000
GA/AA	69	24	60		2.038 (0.954–3.041)
**Chemotherapy Based on CDDP**					
**Genotype**	**Patients, *n* =173**	**Deaths, *n* = 86**	**MST (Months)**	**log-Rank *p***	**HR (95% CI) ^a^**
GG	54	20	N/A^1^	0.023	1.000
GA/AA	119	66	36		1.764 (1.069–2.911)

Abbreviations: HR, hazard ratio; CI, confidence interval; MST, median survival time. ^a^ HR adjusted for age, sex, TNM stage; l-OHP, oxaliplatin; CDDP, cisplatin. N/A^1^, mean the median survival time could not be measured.

**Table 5:** Stepwise Cox regression analysis on the survival of gastric cancer.

Variables	B	SE	HR	95% CI	*p* Value
Age	0.316	0.189	1.371	0.947–1.985	0.094
Sex	0.267	0.228	1.306	0.835–2.042	0.242
Histological types	−0.145	0.193	0.865	0.592–1.264	0.454
Regimes (l-OHP *vs.* DDP)	0.391	0.198	1.479	1.003–2.180	0.048
Dominant model (GG *vs.* GA/AA)	0.17	0.121	1.393	0.929–2.089	0.109

Abbreviations: B, relative risk rate; SE, Standard error; HR, hazard ratio; CI, confidence interval; l-OHP, oxaliplatin; DDP, cisplatin.

## 3. Discussion

In the present study, TNM stage and invasion depth were identified as independent prognostic factors, which is consistent with conclusions from previous studies [24,25,26,27]. Further, we found for the first time that *POU5F1P1* rs10505477 GA/AA genotypes indicated poorer overall survival of gastric cancer in patients undergoing chemotherapy based on CDDP, compared with the GG genotype. This finding had never been demonstrated by other researchers before. However, no association between rs10505477 and survival of gastric cancer in either genotype was observed for oxaliplatin therapy.

Gastric cancer is a stem cell disease [4,5,6,7]; tumor stem cells have been identified with characteristics of pluripotency and self-renewal. Normally, stem cells exist in their own micro-ecological environment, maintaining the stability of the body through proliferation and differentiation. With genetic changes or alteration in the microenvironment, the regulatory mechanisms of stem cell proliferation and differentiation is disrupted, and, as a result, tumors may form [9,28].

*POU5F1*, a member of the *POU* (*Pit-Oct-Unic*) transcription factor family, is one of the most important transcription factors for maintaining the stem cells’ pluripotent and self-renewing state [9,10,11]. *POU5F1* is expressed not only in embryonic stem cells and germ cells but also in various types of solid tumor cells, including gastric cancer [12,13]. It has been confirmed in some reports that *POU5F1* plays an important role in gastrointestinal malignancy through WNT/β-catenin, TGF-β, JAK3/AKT and STAT3/Survivin pathway [29,30,31,32]. *POU5F1P1* gene was classified as a highly homologous pseudo-gene of *POU5F1*. It has been reported that *POU5F1P1* produces a protein with similar function to *POU5F1* and that it is associated with prostate cancer [16,33], breast cancer [17] and colorectal cancer [19,20,21,22], whereas the association of *POU5F1P1* with gastric cancer is poorly understood. In 2011, Paul *et al.* first detected no significant association of rs10505477 with upper gastrointestinal cancer in Caucasians [24]. However, there was no research conducted to investigate if the mutation of *POU5F1P1* rs10505477 is associated with gastric cancer or not in Asians. Thus we performed this study to investigate the correlation of *POU5F1P1* rs10505477 with the survival of gastric cancer patients in a Chinese Han population. We found that the patients with the A allele receiving CDDP-based chemotherapy after gastrectomy had worse prognosis.

The REAL-2 and some other studies [34,35] similarly demonstrated the same result that there was no significant difference between oxaliplatin-containing and cisplatin-containing regimens and Arbeitsgemeinschaft Internistische Onkologie (AIO) trial revealed that oxaliplatin could be substituted for cisplatin [36]. Thus, in this study, patients were stratified by chemotherapy regimens (oxaliplatin-based and cisplatin-based), then the prognosis was analyzed for the different genetic models. The result showed that the A allele was a risk factor for the prognosis of these patients having chemotherapy based on cisplatin. But there was no relationship with the prognosis for rs10505477 and gastric cancer patients receving oxaliplatin-based chemotherapy. Thus, we propose that the rs10505477 genotypes can be a potential predictive biomarker of response to cisplatin-based chemotherapy. As far as we are aware, cisplatin and oxaliplatin are the standard platinum drugs. They share the same basic mechanism of anti-tumor action by influencing their common pharmacological target namely DNA. But their mechanism of antitumor action and drug resistance are not exactly the same. This may be the reason why rs10505477 can predict response to cisplatin but not be related to oxaliplatin. The underlying mechanisms of cisplatin resistance are complicated, including reduced concentration of the drug via efflux pumps and detoxification enzymes, or enhanced DNA repair activity, and so on [37,38]. Previous studies have given evidence that over-expression of AKT [39,40,41,42], activation of the STAT3 [43,44] and Wnt signaling pathways [45,46] and down-regulation of c-Myc expression [47] all contribute to cisplatin resistance. In our study, we found that the polymorphism of rs10505477 was significantly associated with the outcome of GC patients treated with cisplatin. We suspect that rs10505477 variants may lead to cisplatin resistance. If this is the case, the underlying mechanisms not fully explored here need further investigation.

A number of limitations should be addressed in this study. First, we only have data for overall survival of the gastric cancer patients, and lack information on disease-specific survival and relapse-free survival. We estimate that most of the patients died of gastric cancer, but lack definitive data on this outcome; Second, the study samples were 909 Chinese GC patients without matched group, and this may lead to bias. Larger sample sizes studies and case-control studies in different populations are needed in the future; Third, in chemotherapy regimen based on CDDP, we found that compared with GG genotype, overall survival of the patients with GA/AA genotypes is decreased significantly. But we cannot make a conclusion that this mutation induces chemo-resistance for lack of large multicenter clinical trials, thus more studies are needed to be carried out to validate our hypothesis.

In conclusion, our results show that *POU5F1P1* rs10505477 polymorphisms have no overall significant association with the survival time of gastric cancer patients; the A allele is a risk factor of the prognosis for these patients only in the subgroup regimen based on cisplatin. Further investigations are required to confirm these findings.

## 4. Material and Methods

### 4.1. Ethics Statement

All participants included in this study had provided written informed consent and the entire procedure was approved by the Institutional Review Board of Nanjing Medical University (Nanjing, China; register ID number: 201203121; 2 March 2012).

### 4.2. Study Subjects

Study subjects were patients with histopathologically confirmed gastric cancer who had received gastrectomy between January 1999 and December 2006 recruited from Yixing People’s Hospital (Yixing, China). None had received chemotherapy or radiotherapy at any point prior to surgery. Nine-hundred and forty-four formalin-fixed and paraffin-embedded samples were obtained. The end point was overall survival (OS). The survival time was calculated from the date of surgery until death or the end of follow-up in March 2009. Death dates were confirmed by review of death certificates of inpatient and outpatient records or obtained through follow-up telephone calls. Patients alive on the last follow-up date were censored. Clinical and pathological variables including age, gender, tumor size, tumor site, histological type, depth of invasion, lymph node metastasis, distant metastasis, TNM stage and chemotherapy regimens were obtained. The TNM stage classification was evaluated according to the criteria of the American Joint Committee on Cancer (AJCC) in 2010. Lauren’s criteria were used to classify the tumors into intestinal and diffuse type.

### 4.3. Genotyping

Genomic DNA of patients was extracted from paraffin sections of tissues by proteinase K digestion, isopropanol extraction and ethanol precipitation. Genotyping was performed with the SNaPshot method using an ABI fluorescence-based assay allelic discrimination method (Applied Biosystems, Foster City, CA, USA) as described previously [25,26,27]. The sequences of the primers used for multiplexed PCR are F-primer (5'-TGTCAATACTGACTTTGCCCCTTTTC-3') and R-primer (5'-TCACCACTTGTCTATCAAACAGGAAGC-3'). The SNaPshot products were analyzed by using ABI 3130xl genetic analyzer (Applied Biosystems) and the genotypes were determined by GeneMapper Analysis Software version 4 (Applied Biosystems). Genotyping assays were performed by two people independently in a blind fashion. More than 10% of the samples were randomly selected for confirmation, and the results were 100% concordant. Nevertheless, 35 samples failed to be genotyped because of poor DNA quality, which were excluded in further analysis. As a result, 909 gastric cancer patients were included in the final analysis.

### 4.4. Statistical Method

Statistical analyses were carried out by using SPSS version 18.0 (SPSS Inc., Chicago, IL, USA) with a two-sided test. The correlations between rs10505477 SNP and clinicopathologic parameters were estimated by using the Pearson chi-square test for categorical variables and the Student *t* test for continuous data. Kaplan–Meier survival curves and the log-rank test were used to evaluate the associations of clinicopathologic variables or rs10505477 SNP with the prognosis of GC. Unvaried or multivariate Cox proportional hazard models, adjuseded for sex, age and TNM stage, were adopted to estimate the crude hazard ratios (HRs), adjusted HRs and their 95% confidence intervals (CIs). Moreover, Cox stepwise regression analysis was performed to assess the independent impacts of SNP or clinicopathologic features on the overall survival (OS) after adjusting for other covariates, with a significance level of *p* < 0.05 for entering and *p* > 0.10 for removing the respective explanatory variables. All tests were two-sided and *p* < 0.05 was considered statistically significant.

## 5. Conclusions

Our preliminary study indicates, for the first time, that *POU5F1P1* rs10505477 polymorphism has no significant association with the survival of gastric cancer patients. However, the A allele is a risk factor for the prognosis of gastric cancer patients receiving cisplatin-based chemotherapy. Further studies are warranted to investigate the mechanism and to verify our results in different populations.
